# Correlation of *Streptococcus mutans* count in Mother-child Pair of Working and Nonworking Mothers: A Cross-sectional Study

**DOI:** 10.5005/jp-journals-10005-1389

**Published:** 2016-12-05

**Authors:** Priyanka Sharma, Mousumi Goswami, Darrel Singh, Shahid S Massod, Khundrakpam Nganba

**Affiliations:** 1 Postgraduate Student, Department of Pedodontics, I.T.S Dental College, Ghaziabad Uttar Pradesh, India; 2 Head, Department of Pedodontics, I.T.S Dental College, Ghaziabad Uttar Pradesh, India; 3 Reader, Department of Pedodontics, I.T.S Dental College, Ghaziabad Uttar Pradesh, India; 4 Student, Department of Pedodontics, I.T.S Dental College, Ghaziabad Uttar Pradesh, India; 5 Student, Department of Pedodontics, I.T.S Dental College, Ghaziabad Uttar Pradesh, India

**Keywords:** Mother-child pairs, *Streptococcus mutans*, Vertical transmission.

## Abstract

**Purpose:**

To determine the prevalence of *Streptococcus mutans* (MS) in mother-child pairs and to evaluate the correlation in the levels of salivary MS of working and nonworking mothers with that of their children and their associations with other related factors.

**Materials and methods:**

A cross-sectional study was carried out among 100 mother-child pairs residing in New Multan Nagar Colony, New Delhi, India. A total of 50 children with their mothers were included in the working group and another 50 were included in the nonworking group. A questionnaire regarding the feeding habits, oral hygiene habits, daily intake of sugars of the children along with their weaning time was carried out. All mothers and children were clinically examined for recording decayed, extracted, and filled teeth (deft)/decayed, missing, and filled teeth (DMFT), and whole unstimulated saliva was collected and cultured for MS in the laboratory. The data were collected and subjected to statistical analysis using chi-square, Spearman’s correlation, and logistic regression analysis.

**Results:**

The prevalence of salivary MS in the children was 69%. A statistically significant correlation was found between the oral levels of MS in nonworking and working mother-child pairs. Regression analysis showed that those children who feed by bottle for more than 12 months, have daily sweet intake, have sugars in feeding bottle and have higher defts were more likely to have mutans score of 1 or 2.

**Conclusion:**

The mother, working or nonworking, being the primary care provider is the major source of transmission of MS to their child irrespective of the amount of time spent with them.

**How to cite this article:**

Sharma P, Goswami M, Singh D, Massod SS, Nganba K. Correlation of *Streptococcus mutans* count in Mother-child Pair of Working and Nonworking Mothers: A Cross-sectional Study. Int J Clin Pediatr Dent 2016;9(4):342-348.

## INTRODUCTION

The World Health Organization proposed the worldwide prevalence of dental caries in their oral health fact sheet.^1^ The pervasiveness of this disease is an outcome of its highly infectious nature due to the microbial component involved, i.e., *Streptococcus mutans* (MS). It is a known fact that a child’s first contact with the environment is made with family members, specifically the mother. The transmission of *S. mutans* is believed to primarily occur vertically along the mother-child infection route, and a discrete window of infectivity has been suggested around 2 years of age.^2,3^ Early acquisition of MS is contemplated to be a major risk factor in the development of early childhood caries (ECC)^4^ as well as further caries experience.^5,6^ A discrete window of infectivity has been suggested in the age group of 7 to 31 months by Caufield.^7^

Earlier, many authors have studied the interrelationships between MS and dental caries experience among a group of children and their parents representing different racial groups. Both clinical and microbiological data were obtained to determine correlations between parent or grandparent and MS.^8^

Mutans streptococci are a group of related oral bacteria found in all individuals. Studies suggest a strong role of MS in the onset of caries, whereas *Lactobacilli* are responsible for active progression of the cavitated lesion. *Streptococcus mutans* and *Streptococcus sobrinus* are considered to be the major etiological agents responsible for the development of dental caries. *Streptococcus mutans* has been found to be the dominant member of the plaque flora in patients with multiple active carious lesions.^8^

From studies on individual subjects, the occurrence of MS is normally higher than that of *S. sobrinus.* They are preferentially found in supragingival plaque, and mothers are the major reservoirs from which children acquire these organisms. Studies conducted with culture methods demonstrated that the salivary level of MS in children is directly proportional to that found in their mothers. Higher MS counts in mothers indicate higher MS counts in their children and vice versa.^9^

Time spent by children, especially preschoolers, with their mothers may play an important role in MS transmission. Thus, this study was undertaken in 100 mother-child pairs (i) to determine the prevalence of MS in children; (ii) to correlate the MS count in working and nonworking mother-child pairs; and (iii) to rule out any relationship of oral health behavioral variable with MS count and caries status.

## MATERIALS AND METHODS

The present study was conducted in the Department of Pedodontics and Preventive Dentistry at I.T.S. Dental College, Greater Noida. The study protocol was approved by the ethics committee of I.T.S. Dental College. A written informed consent was obtained from the selected participants. hundred total of 100 mother-child pairs residing in Multan Nagar Colony, New Delhi city, were selected for the study.

Our study sample included 100 children who were between 1 and 5 years of age and their respective mothers. Samples were selected by stratified random sampling technique. All mothers belonged to the upper socioeconomic status (Kuppuswamy’s Socioeconomic Status Scale).^10^ The selected sample of children was paired with their mothers. These mother-child pairs were divided into two groups of working and nonworking mothers, comprising of 50 mother-child pair each.

Mothers were instructed not to brush their own or their children’s teeth 2 hours prior to the assessment. At their residence, they were interviewed in accordance with a structured questionnaire involving information about various oral health behavioral variables. The child’s medical history, use of antibiotics, existing oral hygiene routines, dietary habits, the weaning period, use of feeding bottles at night, breastfeeding, and sugar in feeding bottles were all included. The mother-child pairs were examined; decayed, extracted, and filled teeth/ decayed, missing, and filled teeth was recorded; and saliva samples were taken for MS assessment.

Samples having history of antibiotic therapy within the last 3 to 4 weeks, fluoride treatment within the last 2 weeks, and acute or chronic systemic disease with potential oral manifestations were excluded from the study.

The saliva sample from both groups was collected by means of sterile swab sticks, i.e., gamma sterile cotton swab stick (Cosmos Scientific Traders, New Delhi, India) for evaluation of MS count. Unstimulated saliva was collected from the buccal vestibule and the sterile swab sticks were transported to the City X-Ray Lab, Tilak Nagar, Delhi, in transport media (Cosmos Scientific Traders, New Delhi, India). Decayed, missing, and filled surface/decayed, extracted, and filled surface (defs) was calculated separately for both mothers and their children.

In the laboratory, the swabs were placed in 1 mL of 0.5 M phosphate buffer (pH 7) solution prepared by mixing the buffer powder in distilled water and was vortexed for 1 minute. The samples were diluted to 1:10 with phosphate buffer solution and were again vortexed. A 50 L volume of each dilution was pipetted onto the Mitis Salivarius Bacitracin sucrose agar plate (HiMedia Laboratories Pvt. Ltd., Mumbai, India) and evenly distributed using sterile spreaders for the cultivation of MS. The plates were incubated at 37°C for 72 hours. The colonies having crusted glass appearance were identified and the colony-forming units (cfu) on each plate were enumerated for the estimation of MS level in the oral cavity. The appearance of MS colonies was confirmed by using American Type Culture Collection control strains (HiMedia Laboratories Pvt. Ltd., Mumbai, India) of MS.

The proportion of children with detectable MS growth were categorized according to the following criteria: [scores - low (<10^4^ cfu), moderate (10^4^-10^6^ cfu), high (>10^6^ cfu)].^11^ The recorded data were transferred to an Excel sheet, and statistical analysis was carried out using Statistical Package for the Social Sciences (SPSS Inc., Chicago, IL, version 20.0 for Windows). Descrip-tives of continuous variables were reported as mean and standard deviation. Categorical variables were reported as frequencies and percentages. The outcome of interest (dependent variable), i.e., mutans score of children, was dichotomized as score 0 *vs* score 1 or 2. The bacterial numbers were subjected to Mann-Whitney U test and the mother-child relationship was calculated with Spearman correlation coefficient between working and nonworking mother-child pairs. Bivariate analysis between dependent variable and several independent variables (predictors) was conducted using chi-square analysis.

## RESULTS

**Graph 1:**
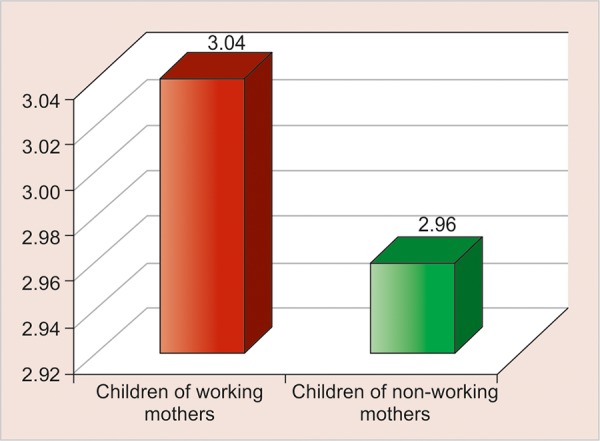
Mean age of children of working and nonworking mothers

A total of 100 children aged between 1 and 5 years were examined. The mean age of children in the working and nonworking groups was 3.04 and 2.96 years respectively (Graph 1).

**Table 1:** **Table 1:** Mean of MS count of working and nonworking mothers and their children

				*Mother’s MS count*				*Child’s MS count*			
*Working* *status*		*n*		mean		Std deviation		mean		Std deviation	
Working		50		1.772 × 10^6^		2.240 × 10^6^		1.859 × 10^4^		1.997 × 10^4^	
Nonworking		50		1.169 × 10^6^		1.980 × 10^6^		8.648 × 10^4^		4.213 × 10^5^	
				p = 0.490, not significant				p = 0.073, not significant			

Mann-Whitney U test

**Table 2:** **Table 2:** Frequency distribution of salivary MS in mothers in scores 0, 1 and 2

		*MS scores of mothers*					
*Working status*		*0 (x < 10^4^)*		*1 (104 < x <* 10^6^)		*2 (x ≥ 10^6^)*	
Non-wrking		2		38		10	
Working		3		27		20	
		p = 0.067, not significant					

Chi-square test

**Table 3:** **Table 3:** Frequency distribution of salivary MS in children in scores 0, 1 and 2 with that of their children

		*MS scores of children*					
*Working status*		*0 (x* < 10^4^)		*1* (10^4^ < *x* < 10^6^)		*2 (x* ≥ 10^6^)	
Nonworking		12		37		1	
Working		19		30		1	
		p = 0.315, not significant					

Chi-square test

**Table 4:** **Table 4:** Spearman correlation coefficient among scores 1, 2, and 3 in working group

*Mothers*				*No of children* * (MS scores)*		*No of children* * (MS scores)*		*No of children* * (MS scores)*	
*MS count*		*n*		*0*		*1*		*2*	
0		3		2		1		0	
1		27		15		12		0	
2		20		2		17		1	

p-value (Chi-square test)        0.017, significant

**Table 5:** **Table 5:** Spearman correlation coefficient among scores 1, 2, and 3 in nonworking group

*Mothers*				*No of children* * (MS scores)*		*No of children* * (MS scores)*		*No of children* * (MS scores)*	
*MS count*		*n*		*0*		*1*		*2*	
0		2		2		0		0	
1		38		9		29		0	
2		10		1		8		1	

p-value (Chi-square test)         0.025, significant

The mean colonization levels of salivary MS in mothers of working and nonworking groups were 1.77 × 10^6^ and 1.16 × 10^6^ cfu respectively. Mean salivary MS count among their children is 1.85 × 10^4^ and 8.64 × 10^4^ cfu in working and nonworking groups respectively (Table 1).

The prevalence of MS in mothers was 95% (Table 2). More than 50% of all mothers in both groups showed moderate count. The prevalence of MS in children was 69% (Table 3). More than 50% of the children in both groups showed moderate MS count.

A very strong positive correlation was found between mother’s salivary mutans count and that of their child among working and nonworking mother-child pairs. This correlation was also found to be highly significant (p < 0.001) (Tables 4 and 5).

Table 6 depicts the prevalence of various oral health behavioral factors in children of working and nonworking groups. There was significant difference in weaning time, bottle-feeding, and breastfeeding among both groups.

Daily toothbrushing was done by 52% of the children of both groups. Thirty percent of the children in the working group used a feeding bottle containing sugar during day, 40% during feeds at night, and 60% had a regular daily intake of sucrose in between meals. In the nonworking group, 30% of the samples had a daily intake of sucrose between meals and 26% of the samples had sugar-containing feeding bottles at night.

**Table 6:** **Table 6:** Distribution of the study population according to oral health behavioral characteristics

*Oral health variables*		*Children of working mothers*		*Children of nonworking mothers*		*p-value*	
Weaning		4-6 months - 46		4-6 months - 33		0.001, significant	
		6-8 months - 4		6-8 months - 17			
Toothbrushing		No - 24		No - 24		0.99	
		Yes - 26		Yes - 26			
Bottle-feeding		6-12 months - 46		6-12 months - 17		0.027, significant	
		12-24 months - 4		12-24 months - 33			
Breastfeeding		<8 months - 48		<8 months - 14		<0.001, significant	
		8-10 months - 2		8-10 months - 34			
		>10 months - 0		>10 months - 2			
Daily intake of sugars		No - 20		No - 35		0.402	
		Yes - 30		Yes - 15			
Sugars in feeding bottle		No - 35		No - 38		0.653	
		Yes - 15		Yes - 12			
Bottle at night		No - 30		No - 37		0.202	
		Yes - 20		Yes - 13			

Chi-square test

**Table 7:** **Table 7:** Association between dental caries of children and their salivary MS counts

		*Children of nonworking mothers*				*Children of nonworking mothers*				*Overall*		*Overall*	
*MS score*		*Defs = 0*		*Defs > 0*		*Defs = 0*		*Defs > 0*		*Defs = 0*		*Defs > 0*	
0		8		4		18		1		26		5	
1		6		31		6		24		12		55	
2		0		1		0		1		0		2	
p-value			0.003, significant				<0.001, significant						
Spearman CC, p-value			0.482, <0.001, significant				0.644,<0.001, significant						

CC, correlation coefficient

**Table 8:** **Table 8:** Bivariate association of various predictors and higher mutans scores

*Oral health variables*		*Children with MS score 0*		*Children with MS score 1 and 2*		*p^a^-value*	
Weaning		4-6 months - 30		4-6 months - 49		0.065, not significant	
		6-8 months - 3		6-8 months - 18			
Toothbrushing		Yes - 15		Yes - 37		0.40	
		No - 18		No - 30			
Bottle-feeding		6-12 months - 21		6-12 months - 25		0.019, significant	
		12-24 months - 12		12-24 months - 42			
Breastfeeding		<8 months - 23		<8 months - 39		0.04, significant	
		8-10 months - 8		8-10 months - 28			
		>10 months - 2		>10 months - 0			
Daily intake of sugars		No - 17		No - 18		0.02, significant	
		Yes - 16		Yes - 49			
Sugars in feeding bottle		No - 15		No - 12		0.007, significant	
		Yes - 18		Yes - 55			
Bottle at night		No - 15		No - 18		0.07	
		Yes - 18		Yes - 49			
Gender		Male - 8		Male - 24		0.265	
		Female - 25		Female - 43			
Mean age of child		3.09 years		2.96 years		0.59^b^	

^a^Chi-square test; independent student’s t test

Weaning started at 4 to 6 months in 92 and 66% children of working and nonworking groups respectively; 96 and 28% children were breastfed till 8 months, whereas only 4 and 68% children were breastfed till 10 months in the working and nonworking groups respectively.

Taking manifest and initial lesions together, caries prevalence was 0.94 ± 0.8 and 1.1 ± 0.7 among the working and nonworking group children respectively. A statistically significant, positive relationship (p < 001) between MS levels and dental caries values was assessed for both groups (Table 7).

Bivariate association in Table 8 depicts significant association in relation to bottle-feeding, breastfeeding, daily intake of sugars, and sugars in feeding bottles between children with low (score - 1) and high (score - 2 and 3) mutans count. Those children who were bottle-fed for more than 12 months, have daily sweet intake, have sugars in feeding bottle, and have higher defs were more likely to have mutans score of 1 or 2. All these three significant independent variables were put into multivariate logistic regression where they were adjusted for all other predictors. Adjusted for all other variables, only defs remained as the statistically significant predictor (Table 9).

## DISCUSSION

Dental caries is a transmissible, pathological infectious disease that decalcifies the hard tissues of the teeth.^12^ It is caused by the interplay of carbohydrate, MS, and tooth in the oral cavity.

In the present study, the overall prevalence of MS was found to be 69% in 1- to 5-year-old children. This is in accordance with a study by Okada et al^13^ where 61.7% prevalence of MS was seen in preschool children, and another study carried out in 3- to 6-year-old children showed the prevalence to be 81%.^12^

The mothers of both groups had higher salivary MS count than their children because salivary MS count has a significant positive corelation with older age, which is in accordance with the present study.^12^

Many studies^7,9,10,14,15^ have reported a positive corelation of salivary MS count with age, except Al-Mashhadani^14^ who reported that salivary MS count was higher in children than adults. Caufield et al,^16^ Antony and Munshi^17^ have previously suggested a quantitative mother-child relationship, although in smaller samples.

**Table 9:** **Table 9:** Logistic regression analysis examining association of various predictors and higher MS scores (score 1 and 2) among children

*Predictor*		*Unadjusted OR*		Adjusted OR**		*95% confidence interval*		*p-value*	
Child age		0.586		-		-		-	
Gender									
Female		1		-		-		-	
Male		1.74		-		-		-	
Mother’s MS score									
MS score 0		1		-		-		-	
MS score 1		8.02		-		-		-	
MS score 2		11.1		-		-		-	
Working status of mothers									
Non		1		-		-		-	
Working		0.436		-		-		-	
Bottle-feeding									
6-12 months		1		1					
12-24 months		2.94*		1.76		0.14-2.87		0.328	
Breastfeeding									
<8 months		1		-		-		-	
8-10 months		2.064		-		-		-	
>10 months		1.8		-		-		-	
Daily intake of sugars									
No		1		1					
Yes		2.89*		1.93		0.61-6.10		0.260	
Sugars in feeding bottle									
No		1		1					
Yes		3.819*		2.31		0.73-7.27		0.151	
Defs		6.266*		6.06		2.53-14.53		0.005	

*p < 0.05; **Adjusted for all other predictors which showed significant association in univariate analysis; OR: Odds ratio

Window of infectivity in primary dentition is between 7 and 31 months, which provides a virgin habitat that enables MS to colonize the oral cavity avoiding competition with other bacteria. *Streptococcus mutans* count was found to be predominant in this period. All the samples of the present study fall in the period of infectivity, hence they are more susceptible to higher colonization of MS.

However, in the present study, highly significant positive correlation (p < 0.001) was found between the MS count of mothers and children of working and non-working group.

In Indian societies, most of the family culture and beliefs dictated that mothers take care of their children on their own. Hence, a child spends maximum time with mother, thereby favoring increased transmission of MS between them. The bacteria present in the mother’s oral cavity gets transmitted by the use of domestic items, such as toothbrushes and spoons contaminated with saliva via sharing utensils, breastfeeding, and kissing.^17^

As a child emerges from the weaning period and approaches and attains school age, it is likely that increasing socialization and broader contacts inside and outside the home are established. But, in today’s time, most of the children like to play on computers, videogames, cellular phones, and other electronic devices available in the market. Hence, these children are more likely to spend most of their time at home in spite of outdoor games.

The working mothers included in this study are mostly teachers whose working timings varied between 8:00 am and 2:00 pm, the time coinciding with the period when the preschoolers are either sleeping or at playschool. Following this, the children spent their whole time with their mothers.

Various other studies have demonstrated the maternal transfer of MS to be as high as 71% in a cohort of Birmingham children, which is in accordance with our present study^18^ and as low as 43% in Toronto, Ontario, children.^19^ Emanuelsson and Bratthall^20^ found 55% maternal transmission and no paternal transmission in Swedish families.

Other studies depict that MS is readily acquired from nonmaternal sources in certain populations. De Soet et al^21^ reported that cleft palate populations receiving obturators early in life demonstrate maternal transmission in only 38% of mother/child pairs. In the Chinese population, Emanuelsson and Wang^22^ reported 36% maternal transmission and 27% paternal transmission.

In another study conducted in a day care setting of Japan, Tedjosasongko and Kozai^23^ evaluated 39 children and found 33% of maternal transmission, 8% of paternal transmission, and evidence of 58% of horizontal transmission from playmates. Mitchell et al^24^ showed maternal transmission was a mode of MS acquisition in 41% (11/27) of mother-child pairs, while acquisition from nonmater-nal sources occurred in 74% of children aged 18 months and 6 years. These studies indicate that, while maternal transmission does occur, it is only one of several modes of MS acquisition. These studies are not in agreement with the present study as it does not include the mutans count of schoolchildren and playmates or their guardians, which can further give us a clear picture of transmission through nonmaternal sources.

Many studies^8,14^ have demonstrated a significant positive relationship (p < 0.01) between MS levels and dental caries values, which was also seen in this study.

It was observed that weaning starts early in working group as these mothers had to leave their children and go out for work. This difference came out to be significant in between two groups also. In bivariate analysis, weaning time and toothbrushing were not related to mutans count. This result is in accordance with a study by Ersin et al^11^ and not in accordance with Habibian et al.^25^

Because breast milk is nutritionally rich, breastfeeding >7 times daily after 12 months of age is associated with increased risk for ECC (American Academy of Pediatric Dentistry guidelines of infant oral health care 14/15). In this study, prolonged bottle-feeding was seen in children of working group, whereas prolonged breastfeeding was seen in children of nonworking group, and this difference was also statistically significant. Similar to some previous studies journal of dentistry for children (JDC), however, the relationship between the duration of breastfeeding and MS counts suggested that prolonged breastfeeding led to higher caries prevalence and allowed the colonization and proliferation of MS and *Lactobacilli* on the teeth of young children, which can be seen in the present study.

Li and Wang^26^ found that children who were breastfed for more than 9 months were likely to harbor strains of MS common to their mothers as compared with children who were breastfed less than 9 months.^24^ This is in accordance with the results of the present study in nonwork-ing group, 72% (34/50) children were breastfed 8 to 10 months, hence harboring more MS count as compared with the children of working group.

Limitation of this study is that the genotype mapping of the MS strains has not been done, which could further show the intrafamilial transfer of MS. Hence, further studies could be done to rule out more descriptive criteria of transmission of mutans and thus help the parents to follow certain preventive measures to reduce the chance of demineralization of the teeth.

## CONCLUSION

At the end of the study, it was concluded that primary caregiver (mother) was the major source of MS transmission to the child. Our study also highlights that if primary caregiver harbors high levels of MS in their saliva, it is likely that their children would also have high levels of MS colonization. Hence, there remains a need for parent counseling and motivation, so that they not only maintain their own oral hygiene but also make a conscious effort to avoid transmission and colonization of these cariogenic bacteria to their children.
